# A Microbiota-Dependent Subset of Skin Macrophages Protects Against Cutaneous Bacterial Infection

**DOI:** 10.3389/fimmu.2022.799598

**Published:** 2022-06-09

**Authors:** Young Joon Park, Byeong Hoon Kang, Hyun-Jin Kim, Ji Eun Oh, Heung Kyu Lee

**Affiliations:** ^1^ Graduate School of Medical Science and Engineering, Korea Advanced Institute of Science and Technology (KAIST), Daejeon, South Korea; ^2^ Department of Dermatology, Ajou University School of Medicine, Suwon, South Korea

**Keywords:** microbiota, Siglec-1, CD169, macrophages, interferon, γδ T cells, IL-17, *S. aureus*

## Abstract

Microbiota is essential to the development and functional maturation of the immune system. The effects of the gut microbiota on myeloid cells remote from the gut, especially the skin remain unclear. Transcriptomic analysis revealed that type I interferon (IFN) signaling was down-regulated in the skin of germ-free mice compared to that in specific pathogen-free mice. The decrease in type I IFN signaling was closely related to the presence of microbiota and macrophage-specific marker CD169. The absence of CD169^+^ macrophages resulted in increased bacterial burden and impaired immune responses against Staphylococcus aureus skin infection. CD169^+^ macrophages mediated the recruitment of γδ T cells as well as the activation of γδ T cells via interleukin (IL)-23. Our findings demonstrate the role of the microbiota in establishment of a specific myeloid cell subset expressing CD169 in the skin and provide evidence of a specific mechanism by which this subset protects against bacterial skin infection.

## Introduction

The microbiome actively affects the host’s immune system in several ways. Commensals residing in the gastrointestinal tract regulate immune responses in the gut and across distal sites other than the gut (1). There are three major mediators of microbiota affecting remote tissues: microbes, microbial products (metabolites), and circulating immune cells (2, 3). These mediators translocate from the intestines *via* circulations and affect immune responses at distal sites. Recently, the gut microbiota was found to influence type I IFN signaling at the remote tissues such as the lung, lymph nodes, and spleen (4, 5).

Type I IFNs and interferon stimulated genes (ISGs) protect the host against viral pathogens, bacteria and parasites (6, 7). Aberrantly expressed type I IFNs and/or ISGs are linked to autoimmune disorders and cancers (8, 9). Type I IFNs are constitutively secreted at low amounts in various organs (10), and the tonic IFN signaling maintains homeostasis of resident immune cells and primes cells to mount a rapid and robust immune response when challenged (4, 10).

Our analysis of a published data suggested that type I IFN signaling increases in the skin of specific pathogen free (SPF) mice compared to germ-free (GF) mice (11). Higher expression of genes related to multiple monocyte/macrophage lineage were also noted in SPF mice. Therefore, in this study, we investigated the possible relationship between increased type I IFN signaling and monocyte/macrophage lineage in the skin. We show that CD169 expression in skin macrophages is regulated by microbiota-dependent type I IFN signaling. Furthermore, we comprehensively explored the characteristics and roles of skin CD169 expressing cells in host defense.

## Results

### Reduced Type I IFN Signaling in GF Skin Alters Expression of the Macrophage-Specific Marker, CD169

**Figure 1:**
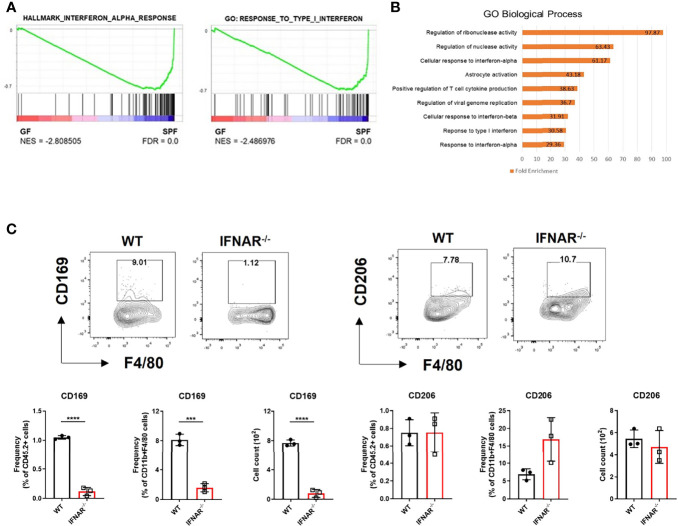
Diminished type I IFN signaling in germ-free skin alters expression of macrophage-specific marker, CD169. **(A)** Gene set enrichment analysis (GSEA) enrichment plots performed on two gene set collections (h.all.v7.2.symbols.gmt and c5.all.v7.2.symbols.gmt) in germ-free (GF) and specific pathogen-free (SPF) mice from data published in Meisel et al., 2018 (GSE98877 from GEO database). GSEA performed 1000 permutations. **(B)** Gene Ontology analysis of GSE98877. **(C)** Representative FACS plot and graphs showing frequency and cell counts of CD169^+^ and CD206^+^ myeloid cells in skin of WT and Ifnar KO mice (n = 3 per group). The cells were gated on FSC/SSC, PI^-^, singlet, CD45.2^+^, CD11b^+^, F4/80^+^ cells. Data represent two independent experiments. Data are expressed as means ± SEM. Horizontal lines above bars indicate statistical comparisons with statistical differences between categories (***p < 0.001, ****p < 0.0001).

We mined the data of a previous study on genes differentially expressed in the skin between GF and SPF mice using the GEO database (11). We pre-ranked 15,448 featured genes using log2FC values and analyzed them using GSEA. GSEA showed increase in type I IFN signature in the skin of SPF mice (**Figure 1A**). Gene Ontology (GO) analysis of the top 100 genes highly expressed in SPF mice revealed an increase in pathways related to type I IFN signaling (**Figure 1B**). There was also selective enrichment of tissue-resident macrophage markers, and monocytes in SPF condition (**Figure S1**). Thus, we hypothesized that there might be a relationship between type I IFN signaling and monocyte/macrophage lineage cells. Macrophage markers were investigated in *Ifnar* KO mice for evidence of this relationship. Mannose receptor CD206 is a well-known macrophage marker of the skin and is important for wound healing (12). CD169^+^ macrophages have been described in skin (13, 14), although their features are not identical to those of CD169^+^ cells in other tissues (15). Both CD206 and CD169, are ISGs, as queried using the Interferome database (16). We speculated that the decrease in type I IFN signaling would affect myeloid cells and result in decreased CD206 and/or CD169 expression. Flow cytometry data showed that CD169 expression was dramatically reduced in CD11b^+^ F4/80^+^ skin cells of *Ifnar* KO mice, but CD206 expression was not (**Figure 1C**).

### CD169^+^ Cells Are Skin Macrophages With Unique Characteristics and Less Frequent in GF Skin

**Figure 2:**
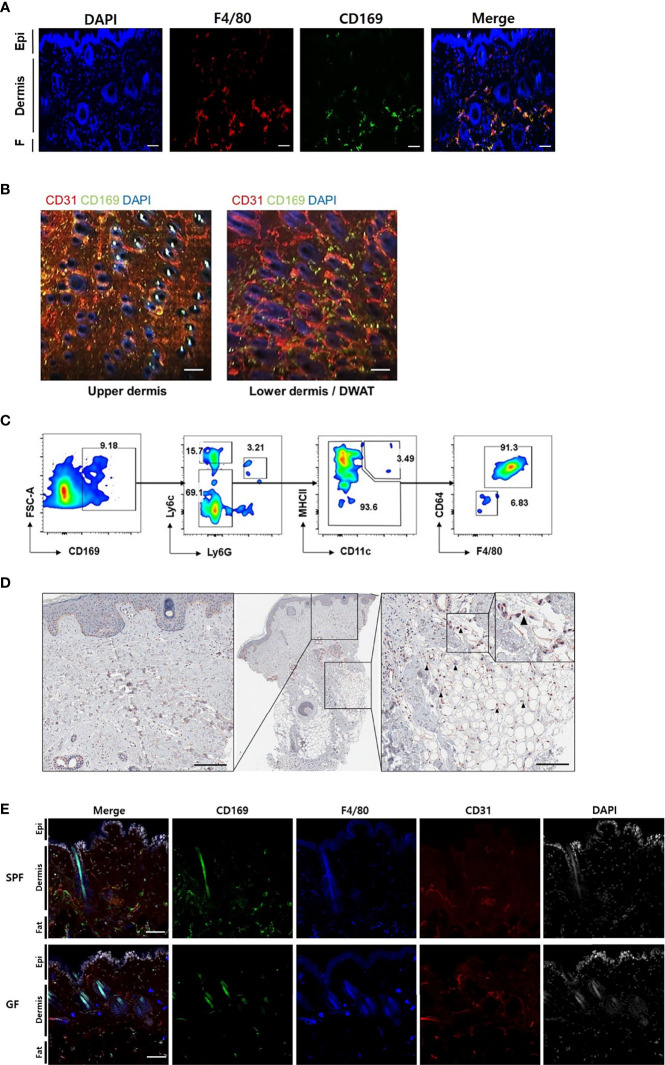
CD169+ cells are detectable in the skin of mouse and human, and less frequent in skin of germ-free mice compared to specific-pathogen-free mice. **(A)** Orthogonal projected image of a vertical skin section (100 µm) using confocal microscopy (F4/80-red, CD169-green, DAPI-blue color). Scale bar indicates 100 μm (Epi: epidermis, F: subcutaneous fat). **(B)** Merged images from a horizontal section (CD31-red, CD169-green, DAPI-blue color) of dermis and dermal white adipose tissue (DWAT) from WT mice. White spots indicate auto fluorescent hair shafts. Scale bar, 100 μm. Images represent three **(A)** and two **(B)** independent experiments. **(C)** Representative gating strategy and features of FACS-sorted CD169^+^ cells in skin. Gated on FSC/SSC, PI^-^, CD45.2 and CD11b. Plots represent two independent experiments. **(D)** Representative human skin sample immunostained with CD169 (black arrows indicate CD169^+^ cells). **(E)** Orthogonal projected images of vertical skin sections (100 µm) from SPF and GF mice (CD169-green, F4/80-blue, CD31-red, DAPI-white color). Scale bar indicates 100 μm. Images represent three independent experiments.

We found CD169^+^ cells primarily in the margins of the lower dermis and subcutaneous fat, otherwise termed dermal white adipose tissue (DWAT) (**Figure 2A**). The cells also overlapped with some F4/80^+^ cells, a marker of myeloid cells. In the horizontal sections, CD169^+^ cells were abundant in the lower dermis and were also mainly perivascular (**Figure 2B**). Most CD11b^+^/CD169^+^ cells were not Ly6c^hi^ monocytes or CD11c^hi^/MHCII^hi^ dendritic cells, but were macrophages, defined by their expression of F4/80 and CD64 (**Figure 2C**). We also found CD169^+^ macrophages in the lower dermis and DWAT of human skin (**Figure 2D**). We presumed that the CD169+ macrophages would be less frequent in skin of GF mice due to its decreased type I IFN signaling. Our confocal microscope images showed lack of CD169^+^ cells in GF skin compared to substantial amount of CD169^+^ cells in SPF skin, as expected (**Figure 2E**). These cells were mostly bone marrow-derived (BMD), as confirmed by domination of CD45.1^+^ and CD169^+^ expressing cells in CD45.1/CD45.2 chimeric mice (**Figure S2A**). High C-X3-C motif chemokine receptor 1 (CX3CR1) expression accounts for tissue-residency and self-renewal with minimal contribution from blood monocytes (17–19). CX3CR1^hi^ macrophages did not overlap with CD169^+^ cells in *Cx3cr1* ^+/gfp^ mice (**Figure S2B**). Nearly all CD169^+^ macrophages were CX3CR1^lo^ (**Figure S2C**). These results indicate that CD169^+^ macrophages were replenished from BMD monocytes.

### Type I IFN Signaling Dictates CD169^+^ Expression in BMDMs and Skin Macrophages

**Figure 3:**
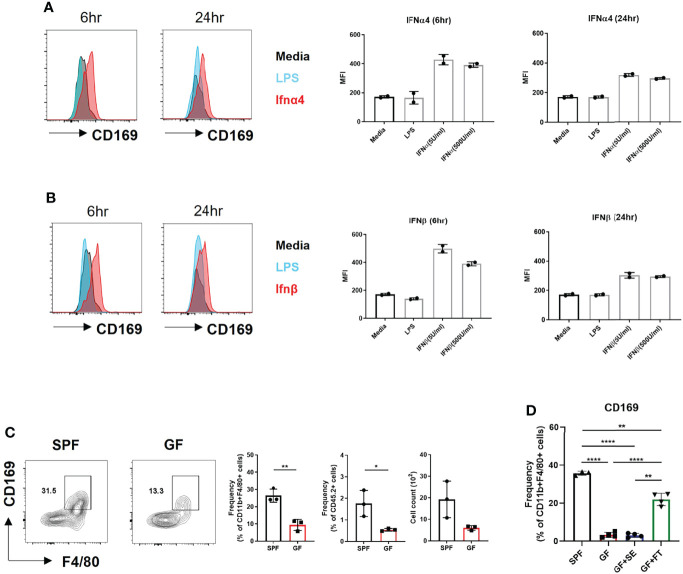
Microbiota-induced type I Interferon signaling dictates CD169^+^ expression in BMDMs and skin macrophages. **(A)** Representative FACS plot and bar graphs showing mean fluorescence intensity (MFI) of CD169 in BMDMs challenged with various doses of recombinant IFNα4 (5 U/mL and 500 U/mL) and **(B)** recombinant IFNβ (5 U/mL and 500 U/mL) at different time points (6 hours and 24 hours). Data represent two independent experiments. **(C)** Representative FACS plot showing frequency and cell counts of F4/80^+^ CD169^+^ cells in skin of SPF and GF mice (n = 3 per group). Gated on FSC/SSC, PI^-^, singlet, CD45.2^+^, CD11b^+^ cells. Data are expressed as means ± SEM (*p<0.05, **p<0.01). Data represent three independent experiments. **(D)** Frequency of CD169^+^ macrophages in skin of SPF mice (n = 3) with DPBS oral gavage (SPF), GF mice (n = 3) with DPBS oral gavage (GF), GF mice (n = 3) with *S. epidermis* topical application (GF+SE) and GF mice (n = 3) with fecal transplantation (GF+FT). The cells were gated on FSC/SSC, PI^-^, singlet, CD45.2^+^, CD11b^+^, F4/80^+^ cells. Combined data of three independent experiments are shown. Data are expressed as means ± SEM (**p < 0.01, ***p < 0.001, ****p < 0.0001).

We investigated whether CD169 expression was induced by type I IFN in BMDMs. IFNα and IFNβ induced higher CD169 expression than media and lipopolysaccharide (LPS) (**Figures 3A, B**). Low levels of type I IFNs was capable of inducing CD169 expression, which was higher at 6 hours post stimulation and decreased after 24 hours. A challenge with a various dose of LPS (10 ng/mL and 100 ng/mL) or *S. aureus* also did not change CD169 expression (**Figure S3A**). The results demonstrate that type I IFN signaling in macrophages might specifically alter CD169 expression, but may not be dose-dependent, due to the change in type I IFN receptor (IFNAR) expression (**Figure S3B**). Our flow cytometry data was in accordance with image data (**Figure 2E**), as CD169 expression was diminished in F4/80^+^ cells of GF mice compared to SPF mice (**Figure 3C**). We speculated that antibiotic treatment might cause a similar decrease in CD169 expression, and we confirmed, albeit statistically insignificant (*p=0.09*), the decrease in skin macrophages (CD11b^+^/CD64^+^ cells) (**Figure S3C**). Finally, fecal transplantation, not topical *S. epidermis* application, was capable of partially restoring CD169 expression in skin macrophages (**Figure 3D**), possibly suggesting the importance of gut microbiota, rather than skin microbiota in CD169 expression.

### CD169^+^ Cell-Deficient Mice Have an Impaired Immune Response Against *S. aureus* Skin Infection

**Figure 4:**
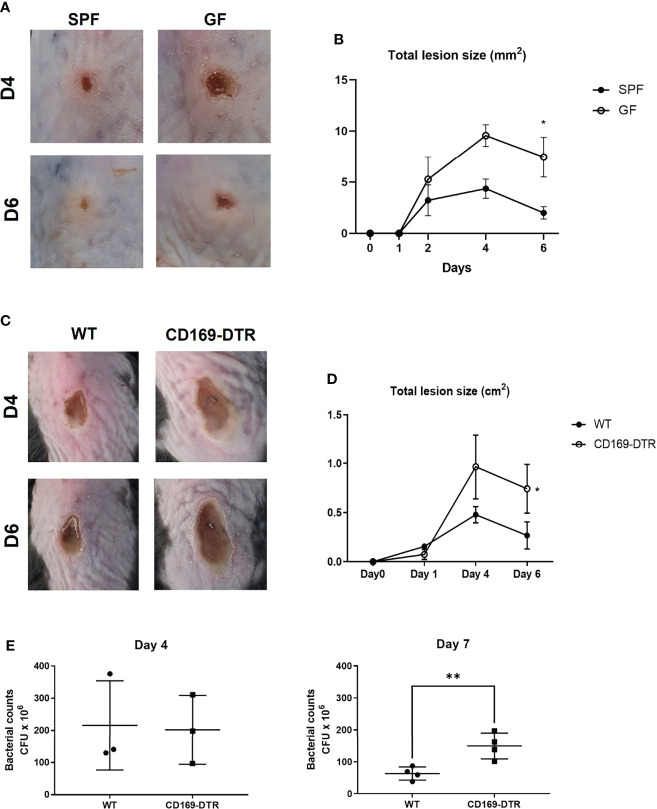
Impairment of immune response in germ-free and CD169^+^ cell-deficient mice against *Staphylococcus aureus* skin infection. **(A)** SPF and GF mice (n = 3 per group) were intradermally challenged with 2×10^7^ CFUs of CA-MRSA (USA300). Representative pictures of skin lesions on day 4 (D4) and day 6 (D6) post infection. **(B)** Total lesion size of SPF and GF mice (n = 3 per group) over-time calculated by ImageJ program. The Images and data represent two independent experiments. **(C)** Representative pictures of skin lesions in WT and CD169-DTR mice (n = 3 per group) after intradermal *S. aureus* infection on D4 and D6 post infection. **(D)** Total lesion size of WT and CD160-DTR mice over-time. The Images **(C)** and data **(D)** represent three independent experiments. **(E)** CFU counts in skin lesion homogenates of WT and CD169-DTR mice after *S. aureus* intradermal infection on day 4 (n = 3 per group) and day 7 (n = 4 per group). Data represent three independent experiments. Data are expressed as means ± SEM (*p < 0.05, **p < 0.01).

**Figure 5:**
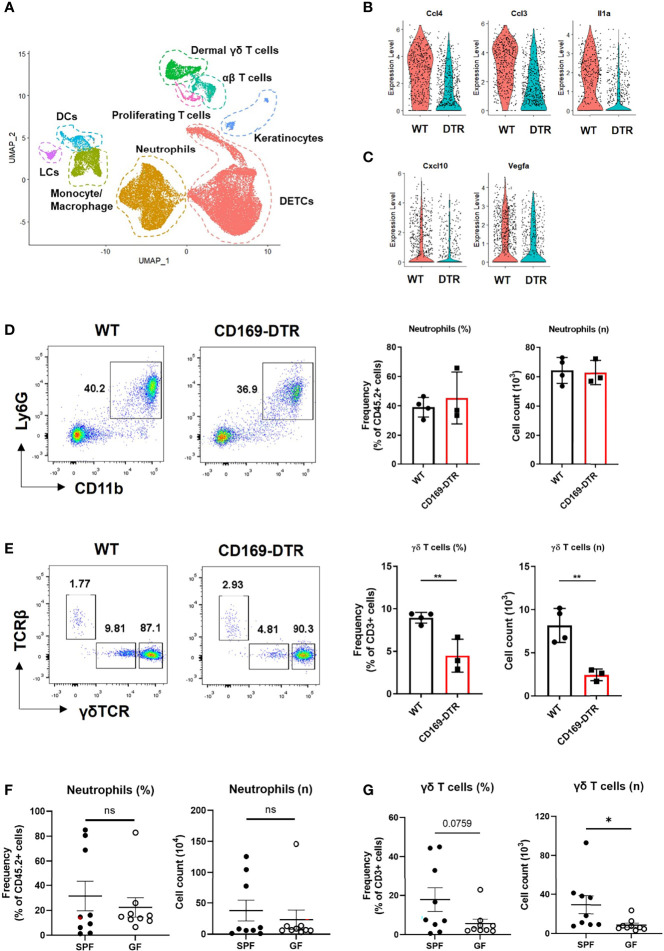
γδ T cells, not neutrophils, are affected by an absence of CD169+ cells at late time points. **(A)** UMAP projections of all skin-infiltrating immune cells. **(B, C)** Violin plot showing DEGs between WT and CD169-DTR of monocyte/macrophage cluster on day 2 **(B)** and day 5 **(C)**. **(D, E)** Flow cytometry analysis of neutrophils **(D)** and dermal γδ T cells **(E)** in WT (n = 4) and CD169-DTR (n = 3) mice skin at day 6 post infection. The cells are gated on FSC/SSC, singlet, PI^-^, CD45.2^+^ **(D)** and FSC/SSC, singlet, PI^-^, CD45.2^+^, CD3^+^, CD11b^-tolo^ **(E)** cells. Data are representative of three experiments. **(F, G)** Flow cytometry analysis of neutrophils **(F)** and dermal γδ T cells **(G)** in SPF (n = 9) and GF (n = 9) mice skin at day 6 post infection. The cells are gated on FSC/SSC, singlet, PI^-^, CD45.2^+^ **(F)** and FSC/SSC, singlet, PI^-^, CD45.2^+^, CD3^+^, CD11b^-tolo^ **(G)** cells. Representative data of two independent experiments are shown. Data are expressed as means ± SEM (*p < 0.05, **p < 0.01, ns, not significant).

To address whether such decrease of CD169+ macrophages might affect host immune response against pathogens, we measured the dermonecrotic area after intradermal challenge with methicillin-resistant *S. aureus* (MRSA) on SPF and GF mice. The size of the skin lesion was significantly larger in GF mice (**Figures 4A, B**). We presumed that the result might, at least in part, be due to decrease of CD169+ macrophages. To further elucidate the role of CD169^+^ macrophages, we used CD169-DTR mice, which have a diphtheria toxin (DT) receptor (DTR) on all cells expressing CD169, and CD169^+^ cells are readily depleted upon administration of DT (20, 21). Mice were treated with DT (25 ng/g bodyweight) on 3 days (D-3) and 1 day (D-1) before *S. aureus* infection. The dermonecrotic area caused by *S. aureus* was significantly larger in CD169-DTR mice compared to WT mice (**Figures 4C, D**). There were significantly more bacteria on day 7 (but not on day 4) in skin of deficient mice of CD169^+^ cells (**Figure 4E**). Taken together, the immune response against *S. aureus* was impaired in absence of CD169^+^ cells. To discern the cause of underlying the difference in immune response, we analyzed isolated immune cells (CD45^+^ cells) on day 0, day 2, and day 5 of *S. aureus* skin infection using scRNA-seq. Using UMAP and conventional cell markers, we determined the lineages of several immune cell clusters (**Figure S4A** and **Figure 5A**). The identified clusters contained fewer infiltrated immune cells in GF mice, confirming the impairment of the local immune reaction (**Figure S4B**).

### γδ T Cell Recruitment Is Impaired in GF and CD169^+^ Cell-Deficient Mice

We analyzed the difference in monocyte/macrophage cluster at each time points with scRNA-seq. The monocyte/macrophage cluster was identified using markers *Ly6c2, Fcgr1*, and *Adgre1*. WT and CD169-deficient mice differed in a number of DEGs on day 2, which included multiple cytokines and chemokines (*Ccl4*, *Ccl3*, *Il1a*, *Cxcl3, Cxcl2, and Cxcl1*) critical to leukocyte recruitment (**Figure 5B** and **Figure S5A**). On day 5, monocyte/macrophage cluster of WT mice did not show any differences between these cytokines and chemokines (**Figure S5B**). *Cxcl10* and *Vegfa* were the only DEGs noted on day 5 (**Figure 5C**). There were no differences in neutrophil number between the two groups on day 6 (**Figure 5D**), but significantly impaired recruitment of γδ T cells (**Figure 5E**), which are known for their involvement in *S. aureus* skin infection (22, 23). Similar significant compromise in recruitment of dermal γδ T cells was noted in GF mice challenged with *S. aureus*, which also did not show differences between SPF mice in neutrophil number on day 6 (**Figures 5F, G**). There were no other significant differences in skin T cells, except decreased frequency of dendritic epidermal γδ T cells (**Figures S5C, D**).

### Activation of γδ T Cells by CD169^+^ Macrophages

**Figure 6:**
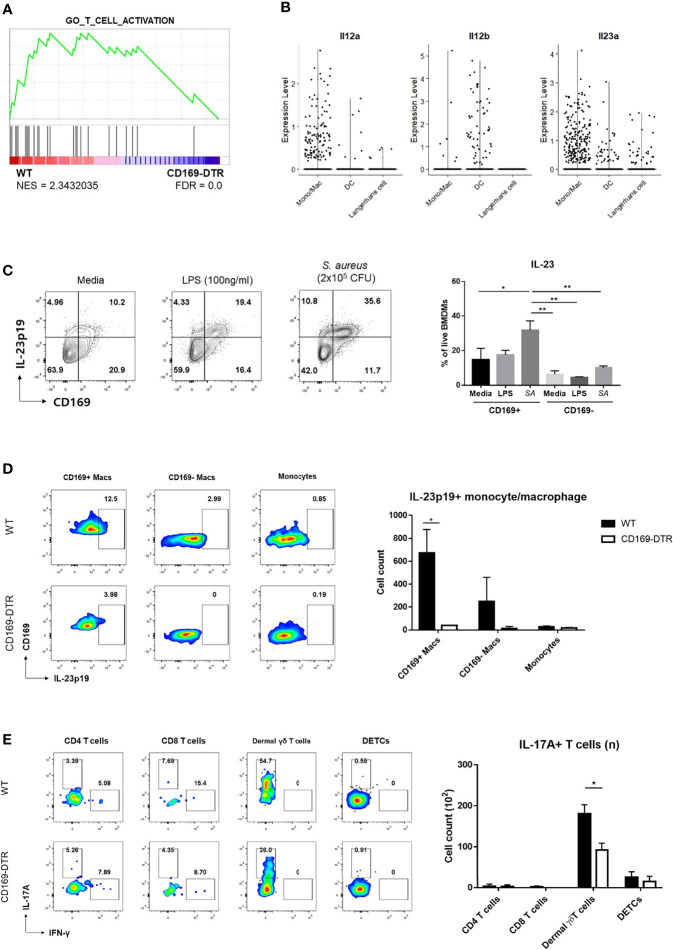
Activation of γδ T cells by CD169+ macrophages. **(A)** GSEA of DEGs from dermal γδ T cells of WT mice compared to CD169-DTR mice. **(B)** Violin plots of Il12a, Il12b, and Il23a expression in monocyte/macrophage, dendritic cell (DCs) and Langerhans cell clusters from scRNA-seq analysis data. **(C)** Representative flow cytometry plots and bar graph of BMDMs challenged with LPS (100 ng/mL) and S. aureus (SA, 2x10^5^ CFU). **(D)** Representative plots and bar graph of IL-23p19 production by CD169^+^ macrophages (Macs), CD169^-^ macrophages and monocytes on day 1 post skin *S. aureus* infection. Single-cell suspensions from two mice were pooled for both WT and CD169-DTR group. CD169^+/-^ Macs were gated on FSC/SSC, PI^-^, singlet, CD45.2^+^, CD11b^+^, EpCAM^-^, Ly6c^-tolo^, Ly6G^-^, F4/80^+^ cells. Monocytes are gated on FSC/SSC, PI^-^, singlet, CD45.2^+^, CD11b^+^, EpCAM^-^, Ly6c^hi^ cells. **(E)** Representative plots and bar graph of IL-17A-producing T cells in skin 6 days post infection. T cells are gated on FSC/SSC, singlet, PI^-^, CD45.2^+^, CD3^+^, CD11b^-tolo^ cells. Single-cell suspensions from two mice were pooled for both WT and CD169-DTR group. Data are representative of three independent experiments. Error bars show mean ± SEM (*p < 0.05; **p < 0.01).

Local activation of γδ T cells was suspected from GSEA of γδ T cells on day 2 (**Figure 6A**) and larger number of IL-17A+ γδ T cells in SPF mice compared to GF mice, despite statistically insignificant (**Figure S6A**). As γδ T cells can be activated without T cell receptor signaling, we investigated whether differences in IL-1β and IL-23 expression by CD169^+^ macrophages resulted in local activation of IL-17-producing γδ T cells. Interestingly, using scRNA-seq analysis, we found that monocyte/macrophage cluster was the most popular source of *Il23a* in myeloid clusters, whereas DCs expressed more *Il12b* (**Figure 6B**). Using BMDMs, we were able to detect IL-23p19 production from macrophages challenged with *S. aureus* and confirmed that CD169^+^ macrophages were the major IL-23p19 producers (**Figure 6C**). Intracellular staining (ICS) of skin immune cells on day 1 post infection revealed that CD169^+^ macrophages mostly produced IL-23p19 production *in vivo* (**Figure 6D**). The absence of CD169^+^ cells resulted in a decrease of IL-17-producing γδ T cells on day 6 (**Figure 6E**). The number of IFN-γ producing T cells was minimal and was not altered in absence of CD169^+^ macrophages (**Figure S6B**). Taken all together, we assumed that the absence of CD169^+^ macrophages result in the decreased number of IL-17-producing γδ T cells, which lead to the impairment of host defense response, as observed from the high CFU counts and a larger area of dermonecrosis.

## Discussion

CD169^+^ macrophages have been studied primarily in lymphoid organs due to their location, for example subcapsular sinus and medullary macrophages in lymph nodes (LNs) and marginal metallophilic macrophages in the marginal zone of the spleen (24, 25). In LNs, the presence of B cells is necessary to generate CD169^+^ macrophages, mediated by their production of lymphotoxin alpha beta (LTα1β2) (26, 27). CD169^+^ macrophages also reside in various peripheral tissues, such as the brain, gastrointestinal tract, liver, lung, kidney, and skin (13, 15, 21). CD169 expression in these tissues is possibly not due to B cells, as peripheral B cells are more infrequent and generally not adjacent to macrophages. Our results suggest that local tonic IFN signaling induced by microbiota might be a factor governing CD169 expression of macrophages in these tissues and yield additional interesting findings. First, CD169-expressing macrophages were present in the lower dermis and within DWAT. Second, these cells are replenished by monocytes. Third, the selective depletion of CD169^+^ macrophages resulted in increased bacterial burden and impaired immune response. This phenotype was possibly due to an impaired ability to recruit γδ T cells. The CD169^+^ macrophages also were capable of γδ T cell activation, which resulted in IL-17 production by γδ T cells. Based on these findings, we concluded that CD169^+^ cells are an indispensable for combating intradermal *S. aureus* infection.

Surprisingly low levels of type I IFNs were sufficient to induce CD169 in BMDMs, while a high amount and/or prolonged type I IFN did not upregulate CD169 expression. CD169 expression might be under the control of type I IFN receptor (IFNAR) expression, which is affected by the strength of type I IFN signaling. Our results, in part, support the notion that a specific tone of type I IFNs is required for homeostatic CD169 expression by macrophages (4, 28). Furthermore, we present the novel mechanism of how microbiota-induced tonic type I IFN signaling affects monocyte/macrophage lineage cells. However, we also found that CD169 expression of skin macrophages *in vivo* is not strictly consistent, which might suggest a possibility of other factors affecting the CD169 expression in more precise manner.

Without CD169^+^ cells, the skin was more susceptible to *S. aureus* infection, as shown by larger dermonecrotic areas and higher CFU counts in CD169^+^ cell-deficient mice than in WT mice. Also, the WT monocyte/macrophage cluster expressed more *Cxcl10* and *Vegfa*, both chemokines important in proliferation phase and remodeling phase of wound healing (29), compared to the CD169-DTR monocyte/macrophage cluster at day 5. We speculated that the lesions of WT mice more successfully eliminated *S. aureus* and were heading towards resolution phase, whereas the CD169^+^ cell-deficient mice remained in the active inflammatory phase. Early excessive neutrophil accumulation, however, might also result in tissue damage and dermonecrosis (30). The area of dermonecrosis should be interpreted along with the data on bacterial burden.

Intradermal injection results in involvement of the whole skin layer including DWAT (31). In this regard, CD169^+^ can act as the initiator of immune responses in intradermal infection, although they are located distantly from the epidermis. A similar role was proposed in a dextran sulfate sodium-induced colitis model, as the colitis symptoms decreased in the absence of CD169^+^ cells (20). The number of RORγt^+^ cells (Th17, γδ T17, LTi, and ILC3) did not change in that study but showed diminished IL-17 and IL-22 mRNA expression. Our data suggested that the skin CD169^+^ macrophages not only affected the recruitment of IL-17A-producing γδ T cells, but also acted as activators of γδ T cells *via* local IL-23 production.

Our study has multiple limitations. First, we did not evaluate the phagocytic activity or previously reported antigen presentation activity of CD169^+^ macrophages (32). It is unlikely, however, that the possible increase in phagocytic activity of CD169^+^ macrophages resulted in such a difference in the phenotype, as the number of resident immune cells were much lower than those of infiltrating neutrophils, monocytes, and T cells. CD169^+^ can also cross-prime CD8 T cells or transfer antigens to CD8a^+^ DCs (32, 33), but acute *S. aureus* infection mostly involves IL-17-producing γδ T cells, in which the activation of the T cells do not require antigen presentation. Consequently, CD8 T cells are likely to have minimal effects on *S. aureus* infection. Second, we were not able to fully elucidate h microbiota enhance type I IFN signaling in skin remain to be elucidated. Previous research suggested that the lung stromal cells (28) and plasmacytoid DCs in lymphatic organs as the source of tonic type I IFN signals (4). These studies indicated that the gut microbiota was involved in the process of tonic signaling, while it is not clear where and how the cells received microbiota signals. We showed that gut microbiota is important in CD169 expression using the fecal transplantation experiment. However, we cannot be completely sure that gut microbiota is entirely responsible for CD169 expression, as not only did we not address the possibility of the act of oral gavage being an inducer of CD169, but also because we were not able to address specific microbiota signals. We also cannot fully exclude the possibility of skin commensals being involved in local type I IFN signaling, as *S. epidermidis* cannot represent the plethora of commensal microorganism skin harbors. Future work will need to explore the exact source and nature of signals leading to enhanced type I IFN and ISG signatures.

## Materials and Methods

### Ethics Statement

All animal experiments in this study were in accordance with the legal and ethical requirements and the guidelines and protocols for rodent research were approved by the institutional animal care and use committee of Korea Advance Institute Science and Technology (KAIST, KA2017-40).

### Mice

SPF male C57BL/6 mice were purchased from the KAIST (Daejeon, Korea) or DBL Co. Ltd (Eumseong, Korea). *Ifnar* knock-out (KO) mice (stock number: 028288, B6(Cg)-Ifnar1^tm1.2Ees^/J) and CX3CR1-GFP (B6.129P2(Cg)-Cx3cr1tm1Litt/J, Stock number: 005582) were purchased from the Jackson Laboratory (Bar Harbor, ME, USA). CD169-DTR (Siglec1<tm1 (HBEGF) Mtka>) mice were purchased from RIKEN BioResource Research Center with the permission of Dr. Masato Tanaka of the Tokyo University of Pharmacy and Life Sciences (Tokyo, Japan). GF mice were obtained from animal care facility of Microbiome Core Research Support Center at Pohang University of Science and Technology (POSTECH, Pohang, Korea). All mice used in this study were maintained in a SPF facility at the KAIST Laboratory Animal Resource Center.

### Bacteria Preparation

Community-associated methicillin-resistant *Staphylococcus aureus* (CA-MRSA) strain USA300 (ATCC^®^ BAA-1717) and *Staphylococcus epidermidis* (ATCC^®^12228) were purchased from the ATCC (ATCC, Manassas, VA, USA) and Korean Collection for Type Cultures (KCTC), respectively. Overnight cultures of a single bacteria colony were pelleted and resuspended in a 15% glycerol solution, aliquoted and stored at -80°C. One hundred microliters of aliquoted bacteria were defrosted and grown overnight in tryptic soy broth (TSB) at 37°C in a shaking incubator (200 rpm). A 2-h subculture (1:20 dilution) was made subsequently. The bacteria were pelleted and resuspended in phosphate-buffered saline (PBS). Optical Density was measured to estimate the colony forming units (CFUs), which was verified after overnight culture on tryptic soy agar (TSA) plates.

### Mouse *S. aureus* Skin Infection Model And Treatments

Mice were anesthetized with 2% isoflurane, and their backs were shaved and depilated a day before *S. aureus* injection. A suspension of 1–2 × 10^7^ CFUs/100μL PBS was injected intradermally using a 29-gauge insulin syringe. Digital photographs of the backs of the injected mice and total lesion size (cm^2^) measurements were analyzed using the image analysis software program ImageJ (https://imagej.nih.gov/ij/). For additional mice treatments, please see the **Supplementary Data**.

### Generation of Bone Marrow Chimeras

C57BL/6 CD45.2 recipient mice were irradiated with 600cGy twice and reconstituted with 5 × 10^6^ BM cells from C57BL/6 CD45.1 donor mice *via* tail vein injection. Chimeric mice were supplied with antibiotic containing water. The transplanted mice were analyzed after 6 weeks.

### Bone Marrow-Derived Macrophage Preparation

Cells were prepared by flushing the femurs and tibiae of mice and were cultured in RPMI-1640 medium supplemented with 10% fetal bovine serum (FBS) (HyClone, Logan, USA), 1% penicillin/streptomycin, and 30% macrophage colony stimulating factor (M-CSF) conditional medium (supernatants of M-CSF from L929 cell cultures). After 7 days, adherent bone marrow derived macrophages (BMDMs) were collected *via* pipetting.

### Enumeration of *S. aureus* CFU

Skin specimens were obtained from the center of lesions using an 8mm-disposable punch (Kai Industries, Seki city, Japan), and homogenized in 1mL PBS using a TissueRuptor II (Qiagen, Germantown, MD, USA). The homogenates were passed through 100-µm cell strainers and plated at serial dilution onto TSA plates. The number of CFU was determined after overnight incubation at 37°C.

### Single Cell Preparation From Skin

Whole skin cells were prepared from mouse back skin. 4 cm^2^ of back skin tissue were harvested. The skin tissue was minced by blade and treated with 2 mg/mL Dispase II (Roche, Basel, Switzerland) for 1 h at 37°C. The skin was further minced into small pieces using scissors and digested with DMEM containing 1% FBS, 1 mg/mL Collagenase IV (Worthington Biochemical Corp., NJ, USA), 0.1 mg/mL DNase I (Roche), and 1.2 mg/ml hyaluronidase (Sigma-Aldrich, St Louis, MO, USA) for 30 minutes at 37°C. The digested skin tissues were then plunged through 70 µm cell strainers. The cells were resuspended on a Percoll gradient (70%/30%) (GE Healthcare, Chicago, IL, USA) and centrifuged at 2000 rpm for 20 min at 25°C. Cells were treated with ACK lysis buffer for 5 min at RT to remove red blood cells.

### Flow Cytometry

Single-cell suspensions were pretreated with anti-CD16/32 (clone 2.4G2; TONBO Biosciences, CA, USA) antibody to block Fc receptors. For additional flow cytometry methods, please see the **Supplementary Data**.

### Single-Cell Transcriptomic Analysis

At 2 days (48 hours) and 5 days (128 hours) after intradermal injection of *S. aureus*, single-cell suspensions were obtained from skin as described above. Cells were treated with anti-CD16/32 to neutralize Fc receptors. Cells then were stained with anti-CD45.2. and 7-AAD, and live leukocytes were isolated using a FACS Aria II cell sorter (BD Biosciences). Single-cell RNA sequencing was performed using a Chromium Single Cell 3’ V3 Reagent kit (10X Genomics, Pleasanton, CA, USA) according to the manufacturer’s protocol. For additional information on single-cell RNA sequencing and its analysis methods, please see the **Supplementary Data**.

### Histological Analysis and Image Acquisition

Skin samples were obtained from the center of the dorsal skin using an 8 mm-disposable biopsy punch (Kai Industries). Samples were fixed in 4% paraformaldehyde (Duksan General Science, Seoul, Korea) and dehydrated in a 30% sucrose solution for 24 h at 4°C. Tissues were embedded in OCT compound (4538, Sakura Finetek, Alphen aan den Rijin, South Holland, Netherlands). Frozen blocks were cut into 100-μm thick sections using a cryostat (Microm HM525, Thermo Fisher Scientific). For detailed information on immunofluorescence staining methods and image acquisition, please check the **Supplementary Data**.

### Analysis of Publicly Curated Gene Expression Dataset

The gene datasets deposited by Grice and Meisel in 2018 were accessed from the Gene Expression Omnibus database (NCBI GEO database, accession GSE98877) (11). The functional classification for the previously published results was analyzed according to their functional similarity based on PANTHER (v15.0, www.pantherdb.org). For GSEA (34), 15,448 differentially expressed genes were annotated to the reference gene set based on MSigDB 7.2.

### Statistical Analysis

All data are presented as means with standard deviations. Statistical analyses were performed using GraphPad Prism 9 (GraphPad Software, Inc., La Jolla, CA, USA) and R statistical program where relevant. An unpaired Student’s t-test was used for analysis between two groups with Welch’s correction when necessary, and unpaired one-way analysis of variance was used for multiple comparisons. A difference between groups was considered significant: *, P < 0.05; **, P < 0.01; ***, P < 0.001.

## Data Availability Statement

Publicly available datasets were analyzed in this study. This data can be found here: https://www.ncbi.nlm.nih.gov/geo/query/acc.cgi?acc=GSE98877.

## Ethics Statement

The studies involving human participants were reviewed and approved by Ajou University Hospital (AJIRB-MED-KSP-21-376). The patients/participants provided their written informed consent to participate in this study. The animal study was reviewed and approved by The institutional animal care and use committee of Korea Advance Institute Science and Technology.

## Author Contributions

YJP, BHK and H-JK conducted the experiments. YJP, JEO and HKL analyzed the data. YJP and HKL wrote the manuscript. HKL supervised the project. All authors contributed to the article and approved the submitted version.

## Funding

This study was supported by the National Research Foundation of Korea (NRF-2021M3A9D3026428 and NRF-2021M3A9H3015688) funded by the Ministry of Science and ICT of Korea.

## Conflict of Interest

The authors declare that the research was conducted in the absence of any commercial or financial relationships that could be construed as a potential conflict of interest.

## Publisher’s Note

All claims expressed in this article are solely those of the authors and do not necessarily represent those of their affiliated organizations, or those of the publisher, the editors and the reviewers. Any product that may be evaluated in this article, or claim that may be made by its manufacturer, is not guaranteed or endorsed by the publisher.
