# Supplementary material for: A Microbiota-Dependent Subset of Skin Macrophages Protects Against Cutaneous Bacterial Infection
Source: Front Immunol. 2022 Jun 9;13:799598. doi: 10.3389/fimmu.2022.799598 (PMC9218056; doi:10.3389/fimmu.2022.799598)
Supplement: Supplementary file 1 [file DataSheet_1.pdf]

Supplementary Data

A microbiota-dependent subset of skin macrophages protects against cutaneous bacterial infection

Young Joon Park[1,2], Byeong Hoon Kang[1], Hyun-Jin Kim[1], Ji Eun Oh[1], Heung Kyu Lee[1]*

[1]Graduate School of Medical Science and Engineering, Korea Advanced Institute of Science and Technology (KAIST), Daejeon, Republic of Korea

[2]Department of Dermatology, Ajou University School of Medicine, Suwon, Republic of Korea

*Corresponding author

Supplementary Methods

Mouse treatments

For antibiotic treatment of mice, 4-week-old mice were administered the following: 100 mg ampicillin, 100 mg vancomycin, 100 mg metronidazole, and 100 mg gentamicin in 100 mL water (changed twice per week) for 4 weeks. For fecal transplantation, a fecal pellet from the control group was homogenized in 1 mL PBS and filtered through a 100-µm strainer; a volume of 200 µL was orally gavaged. Oral gavage was performed on days 0, 2, 4 and 6. For epicutaneous bacteria exposure, approximately 1×10^8 CFUs/100 µL PBS of *Staphylococcus epidermidis* was placed on a sterile gauze pad (1 × 1 cm) and attached to the depilated back of mice using bio-occlusive dressing (Tegaderm, 3M, USA)[1]. The *S. epidermidis* applied gauze pad was changed every other day (days 2, 4 and 6). Cells were harvested from back skin on day 9 post-treatment.

Flow cytometry

The cells were stained with the following antibody mixture of anti-mouse CD3ε (clone 145-2C11), CD11b (clone M1/70), CD11c (clone N418), CD44 (clone IM7), CD62L (clone MEL-14), CD64 (clone X54-5/7.1.1), Ly6C (clone AL-21), Ly6G (clone 1A8) [BD Biosciences, CA, USA], F4/80 (clone: BM8), MHC Class II (clone: M5/114.15.2) [eBioscience, CA, USA], CD4 (clone: GK1.5), CD8α (clone: 53-6.7), CD45.2 (clone: 104), CD169 (clone: 3D6.112), γδ-TCR (GL3) [Biolegend, CA, USA], or CD64 (clone: X54-5/1.1.1) [Invitrogen, CA, USA]. 7-AAD (BD Biosciences) or propidium iodide (Invitrogen) was used to exclude dead cells. Intracellular cytokine staining of T cells was performed based on a previously described method.[1] Briefly, we incubated cells with 50 ng/mL phorbol myristate acetate (Sigma Aldrich), 1 μg/mL ionomycin (Sigma Aldrich), and 2 μM GolgiStop (BD Biosciences) for 5 h at 37 °C. We then stained cells with surface markers prior to fixation and permeabilization using the Cytofix/Cytoperm kit (BD Biosciences) according to the manufacturer's protocol. Anti-IFN-γ (XMG1.2, Biolegend) and interleukin (IL)-17A (TC11-18H10, BD Biosciences) were used to detect intracellular cytokines. All stained samples were analyzed using an LSR Fortessa cell analyzer (BD Biosciences). All flow cytometry data were analyzed using FlowJo software (Tree Star, OR, USA).

Single-cell transcriptomic analysis

Next-generation sequencing was performed using an Illumina HiSeq X Ten System (San Diego, CA USA) for 10,000 cells per sample. Sequencing results were converted into FASTQ files using Cell Ranger (10X Genomics). Samples were aligned using the mouse genome 10-3.0.0 (10X Genomics) as a reference. Matrices were loaded into Seurat (v.3) for data analysis, and R (version 3.6.1) was used for statistical analyses. Cells with an RNA count too low < 200 or mitochondrial gene expression too high (> 15%) were excluded. Data were normalized using the NormalizeData function. Variance of gene expression was checked using the FindVariableFeature function.

Datasets for the control and CD169+ cell-deficient groups were integrated using the FindIntegrationanchors and IntegrateData functions. Data dimensions were reduced using principal component (PC) analysis (PCA) to 20 significant PCs for each sample. Data were clustered using the FindNeighbors and FindClusters functions at various resolutions. RunUMAP was used to visualize the selected PCs. Data were aligned and normalized for the number of genes per cell and mitochondrial gene content using the Seurat workflow[2]. Cluster markers were identified using the FindMarkers function.

Histological analysis and image acquisition

Tissues were blocked with 0.3% Triton X-100 in DPBS with 5% goat serum. Samples were stained with anti-CD169, anti-CD31 and/or anti-F4/80. After washing three times, samples were stained with FITC-conjugated anti-Armenian Hamster IgG (127-025-099, Jackson), rhodamine-conjugated goat anti-Rat IgG (127-025-003, Jackson), and Cy5-conjugated anti-Rabbit IgG (111-175-144, Jackson). The samples were mounted with medium containing DAPI (ab104139, Abcam). Images were obtained by confocal microscopy (LSM800, Zeiss, Oberkochen, Germany) and processed with Fiji (https://fiji.sc/)[4]. For human skin histology, we used human skin samples from normal skin biopsies of patients at Ajou University Hospital, with written, informed consent (AJIRB-MED-KSP-21-376). For image acquisition of histology and immunohistochemical staining, we used a ScanScope CS system (Aperio Technologies, Inc., Vista, CA, USA) to image previously sectioned skin tissue stained with hematoxylin and eosin.

References

1. Liu H, Archer NK, Dillen CA, Wang Y, Ashbaugh AG, Ortines RV, et al. Staphylococcus aureus Epicutaneous Exposure Drives Skin Inflammation via IL-36-Mediated T Cell

Responses. Cell Host Microbe. 2017; 22:653-66 e5

2. Oh JE, Kim BC, Chang DH, Kwon M, Lee SY, Kang D, et al. Dysbiosis-induced IL-33 contributes to impaired antiviral immunity in the genital mucosa. Proc Natl Acad Sci U S A. 2016; 113:E762-71.
3. Stuart T, Butler A, Hoffman P, Hafemeister C, Papalexi E, Mauck WM, 3rd, et al. Comprehensive Integration of Single-Cell Data. Cell. 2019; 177:1888-902 e21.
4. Schindelin J, Arganda-Carreras I, Frise E, Kaynig V, Longair M, Pietzsch T et al. Fiji: an open-source platform for biological-image analysis. Nat Methods. 2012; 28;9(7):676-82.

Supplementary Figures

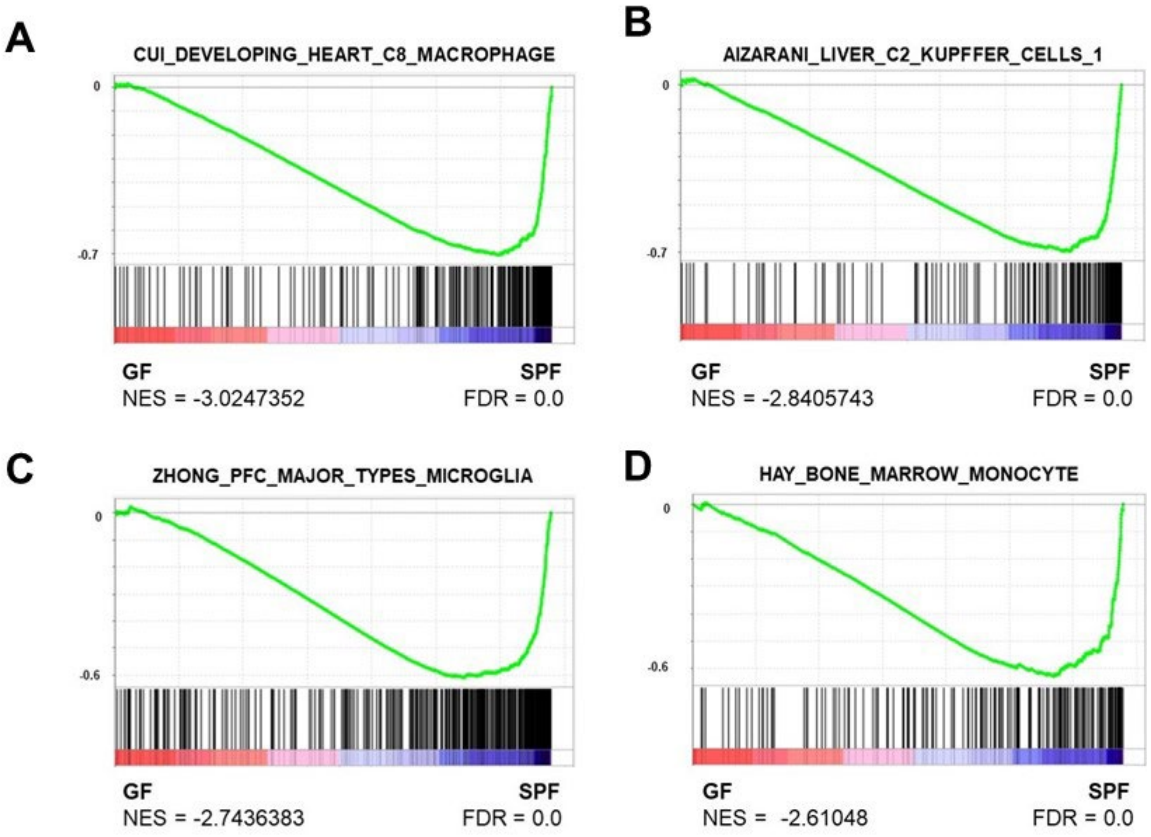

Fig. S1. GSEA showing multiple cell signatures related to tissue-resident macrophages and monocytes.

(A, B, and C) Tissue-resident macrophages. **(D)** Bone marrow monocytes. The c8.all.v7.2.stymbols.gmt gene sets database was used as the gene set collection for analysis of 15,448 differentially expressed genes (DEGs). GSEA performed 1000 permutations.

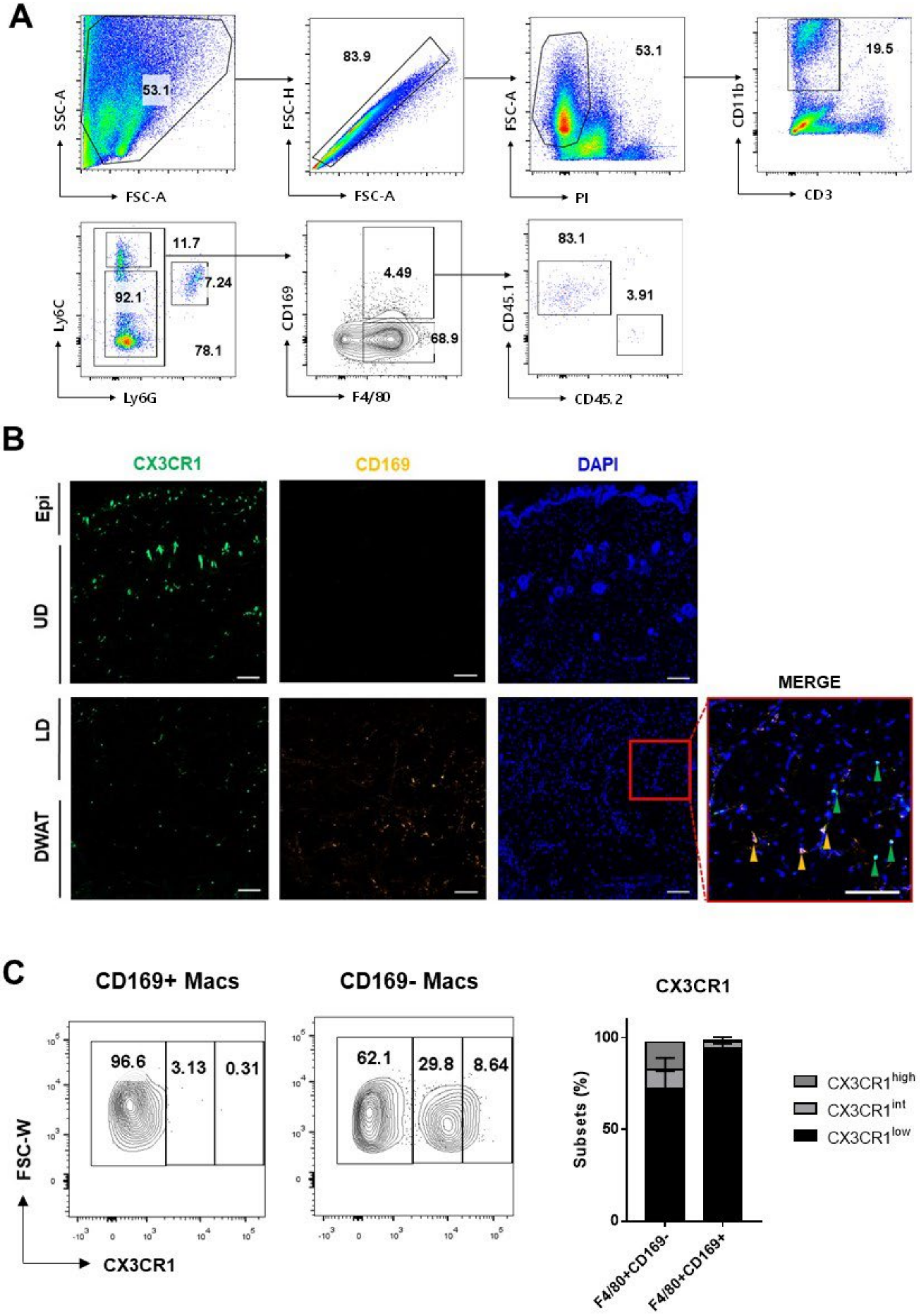
A
SSC-A
FSC-A
53.1
FSC-H
83.9
FSC-A
PI
53.1
CD11b
CD3
19.5
Ly6C
Ly6G
11.7
7.24
92.1
78.1
CD169
F4/80
4.49
68.9
CD45.1
CD45.2
83.1
3.91
B
CX3CR1
CD169
DAPI
Epi
UD
LD
DWAT
MERGE
C
CD169+ Macs
CD169- Macs
FSC-W
CX3CR1
96.6
3.13
0.31
62.1
29.8
8.64
CX3CR1
Subsets (%)
F4/80+CD169-
F4/80+CD169+
CX3CR1^high
CX3CR1^int
CX3CR1^low

Fig. S2. CD169+ cells are CX3CR1^{lo} macrophages replenished by circulating monocytes.

(A) Gating strategy and percentage of CD45.1 and CD45.2 CD169+ macrophages from a mixed BM chimeric mouse. (CD45.1: WT and CD45.2: WT) **(B)** Orthogonal projected image of a vertical skin section (100 µm) using confocal microscopy. Green and yellow arrows indicate CX3CR1-GFP+ cells and CD169+ cells, respectively. Images represent two independent experiments. Scale bar indicates 100 µm. (Epi: epidermis, UD: upper dermis, LD: lower dermis, DWAT: dermal white adipose tissue, CX3CR1-green, CD169-yellow DAPI-blue) **(C)** Representative FACS plots and graph showing CX3CR1 expression in CD169+ and CD169- macrophages (Macs). The suspended single cells were gated on FSC/SSC, singlet, PI-, CD45.2+, CD11b+, Lin*-, CD11c^{-tolo}, MHCII^{-tolo}, Ly6-, F4/80+ cells. *Lineage markers consist of CD3e, NK1.1, Ly6G and B220. Data are representative of two independent experiments. Error bars show mean ± SEM.

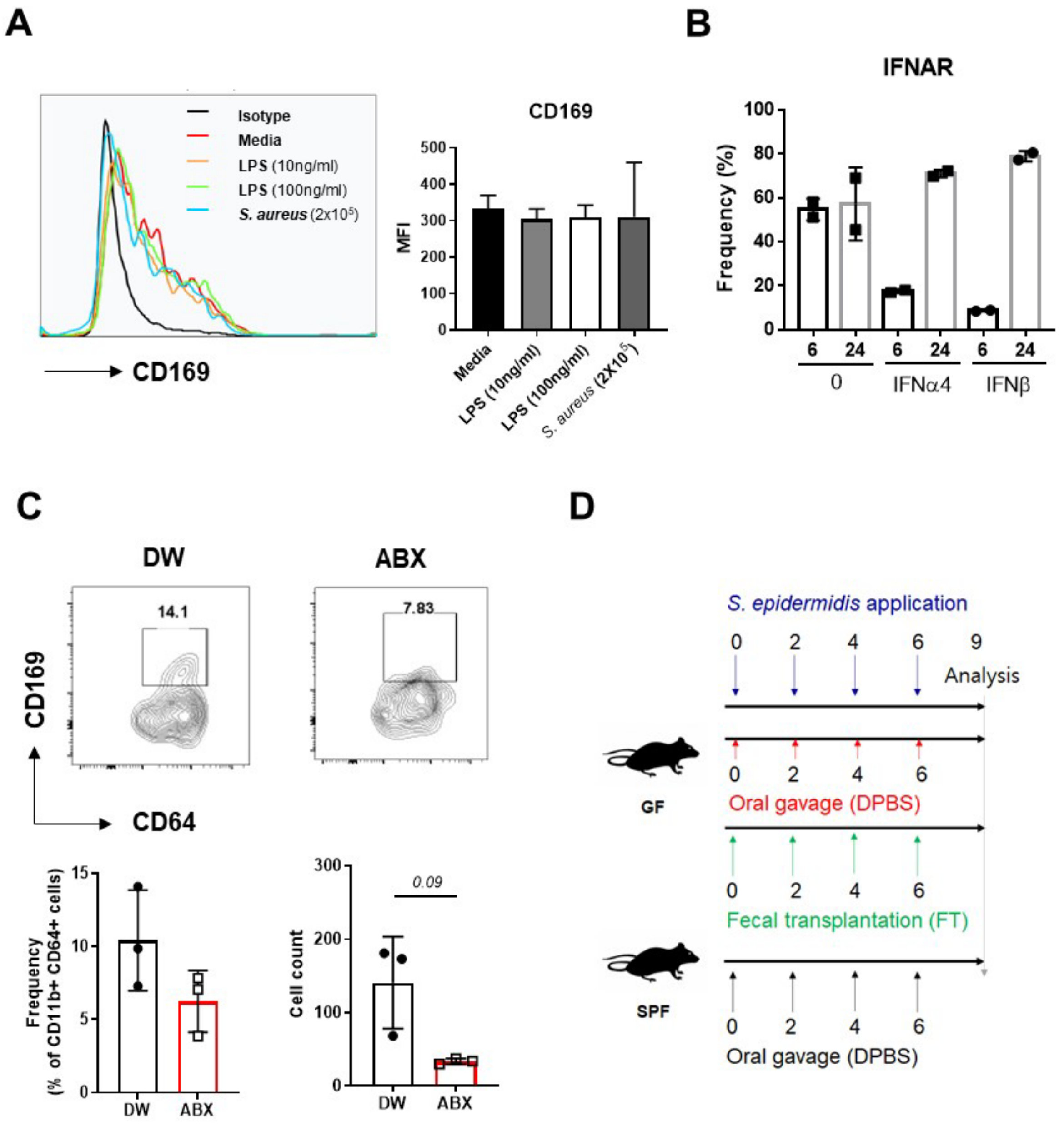

Fig. S3. CD169 expression in BMDMs and skin macrophages.

(**A)** Representative FACS histogram plot and bar graph of mean fluorescent intensity (MFI) values challenged under bacterial stimulants. **(B)** Frequency of IFNAR expressing BMDMs at different time points. BMDMs were challenged with recombinant IFNα4 (5 U/mL) or IFNβ (5 U/mL). (**C)** C57BL/6 mice were administered ampicillin, vancomycin, neomycin sulfate, gentamicin, and

metronidazole (ABX) in drinking water or were given distilled water (DW) as a control for 4 weeks. Representative FACS plot and bar graph showing CD169 expression in macrophages and total cell count of CD169+ macrophages. Gated on FSC and SSC, PI-, CD45.2+, CD11b+ and CD64+. Data are expressed as means ± SEM (*$P < 0.05$). Data are representative of two independent experiments. **(D)** Experimental scheme of mice treatments including oral gavage with DPBS, fecal transplantation and *S. epidermidis* application.

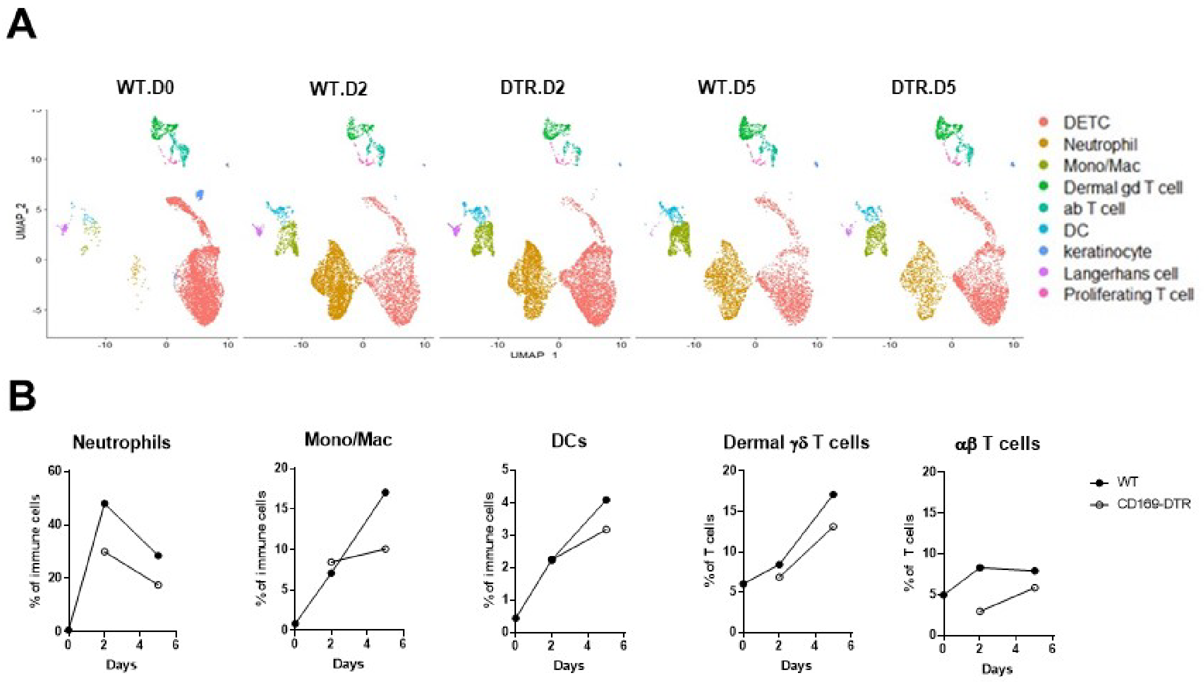

Fig. S4. ScRNA-seq reveals diminished immune cell infiltration in absence of CD169+ cells.

(A) UMAP projections of all skin-infiltrating immune cells from all samples. DETC: dendritic epidermal T cell, Mono/Mac: monocyte/macrophage, Dermal γδ T cell: dermal γδ T cell, ab T cell: αβ T cell, DC: dendritic cell. (**B)** Abundance of each immune cell cluster identified using conventional cell markers of WT and CD169-DTR mice on days 0, 2, and 5.

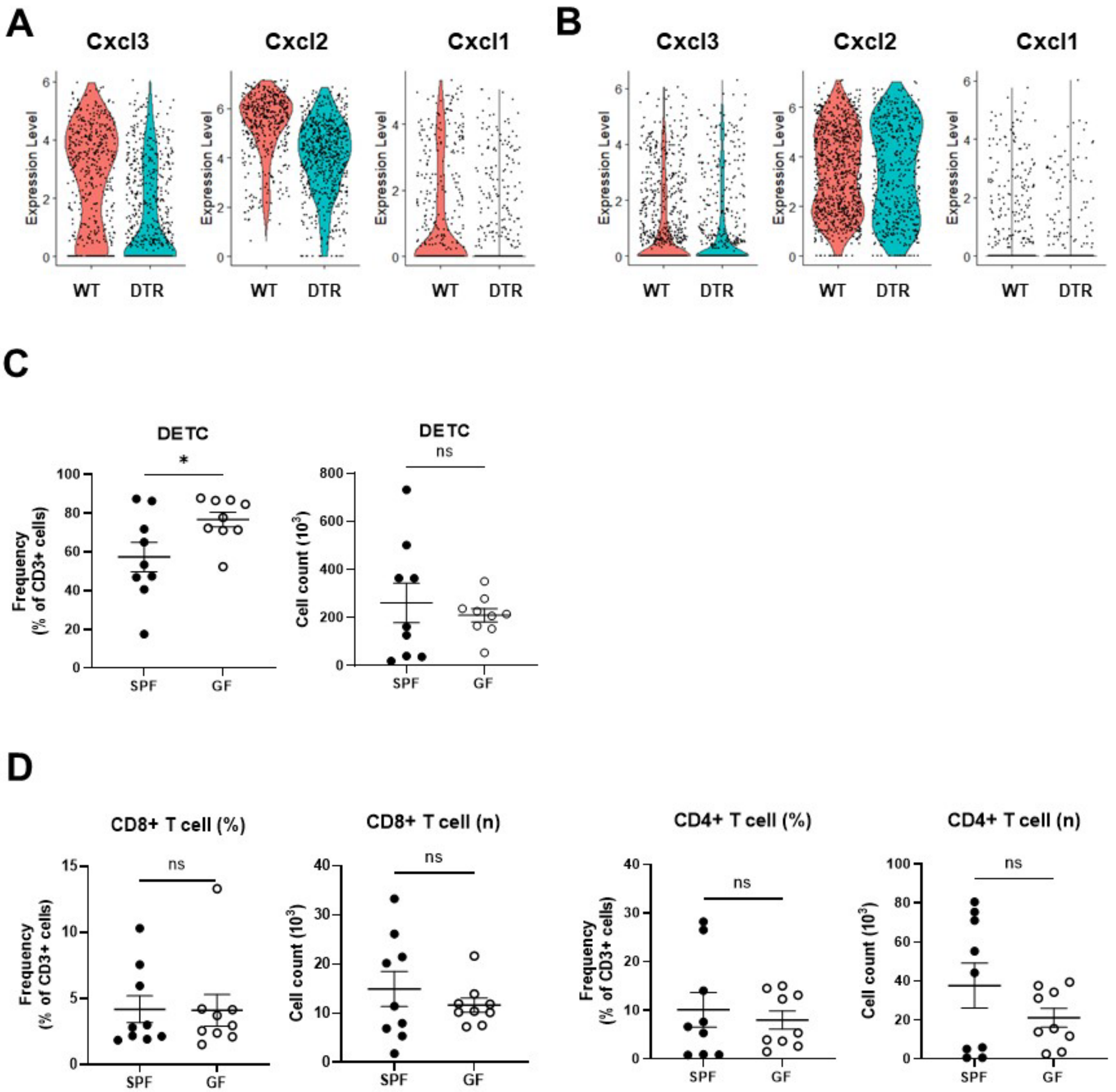

Fig. S5. Neutrophil and γδ T cell recruitment is impaired in CD169+ cell-deficient mice at early time points.

(A-B) Violin plots of *Cxcl1, Cxcl2* and *Cxcl3* between WT and CD169-DTR (DTR) monocyte/macrophage clusters identified from scRNA-seq analysis of **(A)** day 2 post infection and **(B)** day 5 post infection. (**C and D)** Flow cytometry analysis of DETCs **(C)**, CD4 and CD8 T cells **(D)** in skin of SPF and GF mice (n = 9 per group) at day 6 post infection. The cells are gated

on FSC/SSC, singlet, PI-, CD45.2+, CD3+, CD11b-tolo cells. Representative data of two independent experiments are shown.

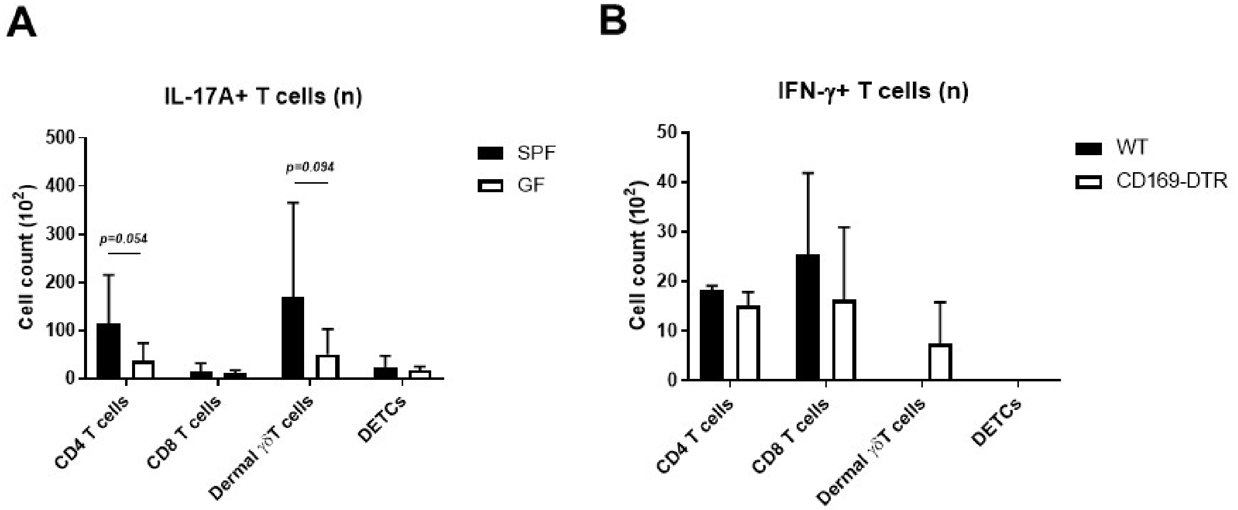

Fig. S6. IL-17A-producing T cells are decreased in GF mice while IFN-γ-producing T cells are not affected by the absence of CD169+ macrophages.

(A) Bar graph of IL-17A-producing T cells in skin of SPF and GF mice 6 days post infection. CD4+ and CD8+ cells are gated on FSC/SSC, singlet, PI-, CD45.2+, CD3+, CD11b^{-tolo} cells. γδ T cells (γδTCR^{int} and CD3^{int}) and DETCS (γδTCR^{hi} and CD3^{hi}) are gated on FSC/SSC, singlet, PI-, CD45.2+, CD3+, CD11b^{-tolo} c, CD4-, CD8- cells. Representative data of two independent experiments are shown. **(B)** Bar graph of IFN-γ-producing T cells in WT and CD169-DTR mice skin 6 days post infection. Single-cell suspensions from two mice were pooled for both WT and CD169-DTR group. Data represent two independent experiments. Error bars show mean ± SEM.
